# Real-Time Data Collection Utilizing Mobile Technology in Clinical Research: A Secondary Analysis of a Prospective Cohort

**DOI:** 10.7759/cureus.72504

**Published:** 2024-10-27

**Authors:** Jessica V Baran, Jerry M Brown, Luke Lamos, Jared Florio, Jesse Beacker, Lea V Oliveros, Abigail L Fabbrini, Andrew A Farrar, Panida Sriaroon, Michael J Wilsey

**Affiliations:** 1 Pediatric Gastroenterology, Johns Hopkins All Children's Hospital, St. Petersburg, USA; 2 Pediatrics, University of South Florida Morsani College of Medicine, Tampa, USA

**Keywords:** barriers to research, clinical research, data collection software, mobile apps (mhealth), mobile phone application, technology

## Abstract

Introduction: Clinical research faces the challenge of declining physician participation in the pursuit of advancing evidence-based medicine. This secondary analysis focuses on the interactive mobile health (mHealth) application’s utility as a real-time data collection tool in clinical settings, specifically targeting cow's milk protein allergy (CMPA) management. The study assesses the mHealth application’s potential to alleviate data collection inefficiencies and improve physician engagement in clinical research.

Methods: The analysis utilized de-identified survey data from a prospective cohort of 61 physicians who employedan interactive mobile survey application over 12 weeks, documenting 808 patient visits.

Results: Of these physicians, 52 (85%) completed initial and follow-up surveys, with the predominant reason for exclusion being incomplete or non-submission of data. Of the 404 patient surveys collected, 75 (19%) were excluded primarily due to the absence of follow-up information.

Conclusion: These results underscore the application’s practicality in streamlining clinical data collection, evidenced by the high rate of survey completion and the efficiency of data management among participating physicians. The findings indicate that interactive mobile health applications aided in collecting and managing clinical data, with 85% of physicians completing surveys for initial and follow-up visits. This high completion rate suggests the potential for mobile applications to mitigate traditional barriers to physician participation, such as time constraints and complex data management. The study contributes empirical evidence to the potential of mobile technology in enhancing research efficiency and engagement among physicians in the context of CMPA management. While the results are insightful, further studies are encouraged to extend the utility of the interactive mobile survey application and similar technologies across diverse clinical research areas, reinforcing mobile technology’s role in transforming clinical research practices.

## Introduction

Mobile devices have become integral to daily life, profoundly impacting healthcare through the development of mobile phone applications or mobile health (mHealth), defined by the WHO as “medical and public health practice supported by mobile devices, such as mobile phones, patient monitoring devices, personal digital assistants, and other wireless devices” [1,2]. These applications have demonstrated potential in various healthcare domains, including fitness, chronic diseases, mental health, and general health monitoring [2-4]. With the emergence of over 100,000 new health mobile phone applications in 2015 alone, the potential of mHealth to enhance medical and public health practice is becoming increasingly evident [2]. However, despite this promising trend, the field of clinical research, a vital component of evidence-based medicine, confronts significant challenges, particularly in engaging physicians

Clinical trials, the cornerstone of evidence-based medicine, are experiencing a concerning decline in physician participation [5-8]. Over the last 40 years, clinical research conducted by practicing physicians diminished from a peak of 4.7% in the 1980s to 1.5% in 2019 [9]. This decline is attributed to factors such as the cumbersome nature of clinical research, financial disincentives, and a lack of available mentors for emerging physician-scientists [9]. Moreover, traditional data collection methods in these trials often posed barriers, including time-intensive processes, complexity, and concerns over data privacy and accuracy [9,10]. The result of these challenges is seen in the low response rates of clinicians in different studies [11-14]. Therefore, there is a pressing need for efficient, user-friendly data collection methods that can help mitigate these barriers and facilitate physician engagement in clinical research. 

This study evaluates a mobile health application as a tool for improving clinical data collection efficiency and physician participation in clinical research, addressing the critical barriers identified in previous research. We conducted a secondary analysis of a mobile application as a real-time data collection tool in clinical research settings within a prospective cohort study involving infants diagnosed or suspected of having cow's milk protein allergy (CMPA). These patients were managed with an extensively hydrolyzed formula (EHF) or an amino acid formula (AAF). The primary goal of this study is to assess the potential of mobile applications in collecting and managing patient data, with a particular focus on evaluating its potential to streamline data collection processes, thereby enhancing clinical research and addressing some of the traditional barriers impeding physician participation. We hypothesize that the use of mobile health applications will streamline the data collection process for clinicians engaged in office-based clinical research.

## Materials and methods

Study objective

This secondary analysis of a primary prospective cohort study evaluated a mobile research application’s effectiveness in real-time data collection from U.S. physicians during patient interactions and its potential to boost physician engagement in clinical research. The initial study involved de-identified data collection at the start of hypoallergenic formula use (baseline) and three to six weeks later (follow-up) in infants six months old or less with suspected or diagnosed CMPA. The findings from the primary study are reported separately [15-18].

Johns Hopkins All Children's Hospital Institutional Review Board issued approval IRB00279920. This study utilized de-identified data and informed consent was obtained by the parents of all patients.

Study population and design

The ZS Moments™ application, developed by ZS Associates (Bellevue, WA, USA) and used in this prospective cohort study, facilitated real-time data capture in a clinical setting with an efficient interface and patient de-identification for privacy. Physicians involved in the study were required to install the application on their mobile devices and complete an initial demographic survey to confirm their eligibility for participation (Table 1). Physicians were recruited in the United States between July and October 2021. The overall study size was determined to be the total number of physicians who met all inclusion criteria by the end of the enrollment period.

**Table 1:** Physician inclusion and exclusion criteria. CMPA: cow's milk protein allergy; EHF: extensively hydrolyzed formula; AAF: amino acid formula

Physician Inclusion Criteria	Physician Exclusion Criteria
Two or more years of experience in a clinic-based setting	Did not treat at least 2 out of the last 10 CMPA patients with a hypoallergenic formula (EHF or AAF)
General pediatrician or specialty in pediatric gastroenterology, pediatric allergy/immunology, or gastroenterology	Switched patient treatment before a subsequent follow-up visit
Seen at least two newly diagnosed CMPA patients per week	

The survey asked physicians about their primary specialty, years in practice, practice setting, average weekly patient count, weekly CMPA patient count, and CMPA treatment plans. After the initial survey, physicians were notified of their study eligibility, and upon confirmation, de-identified data was collected during patient encounters. Each patient was assigned a unique animal identifier to maintain anonymity across visits and to reduce statistical bias. Patient eligibility criteria are detailed in Table 2.

**Table 2:** Patient inclusion and exclusion criteria. CMPA: cow's milk protein allergy; EHF: extensively hydrolyzed formula; AAF: amino acid formula

Patient Inclusion Criteria	Patient Exclusion Criteria
Diagnosed or suspected CMPA	Did not receive a hypoallergenic formula (EHF or AAF) as treatment
Treated with a hypoallergenic formula (EHF or AAF)	>6 months of age at the time of treatment
≤6 months of age at the time of treatment	Did not have survey data collected at the start of treatment and/or at the next follow-up visit
Survey data was collected at the start of treatment and the subsequent follow-up visit (at least three weeks later)	

**Figure 1:**
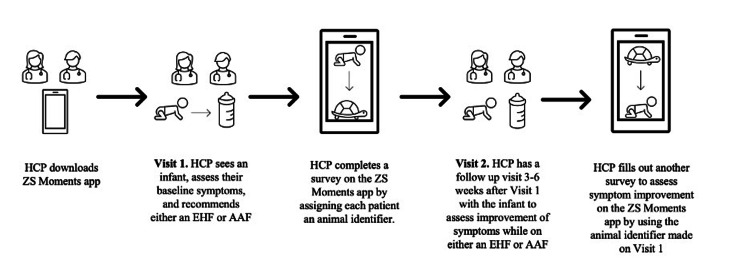
Pictograph of survey timeline. Image Credits: Jessica V. Baran HCP: healthcare professional; EHF: extensively hydrolyzed formula; AAF: amino acid formula

The initial survey collected details about the visit type (in-person or telemedicine), patient demographics (age, gender, height, and weight percentiles), family allergy history, and current symptom severity, rated from 0-3. The follow-up survey, after prescribing hypoallergenic formula, recorded changes in treatment, symptom scores, and physician’s satisfaction with the CMPA treatment, including its effectiveness and recommendation potential. A pictograph of the study timeline appears in Figure 1.

Statistical analysis

Statistical analysis was conducted to assess the differences in patient outcomes between those using EHF and AAF. Descriptive statistics were employed to summarize the physician and patient demographics, as well as the frequency of patient management by the participating physicians. Categorical variables, such as physician specialty and patient demographics, were presented as frequencies and percentages, and comparisons between groups were made using chi-square tests. For continuous variables, such as the duration between baseline and follow-up visits, mean values and standard deviations were calculated, and independent t-tests were used to compare the means between the EHF and AAF cohorts. All analyses were performed using SPSS® software (IBM®, Armonk, NY, USA), with a significance level set at p < 0.05.

## Results

Physician demographics

**Figure 2:**
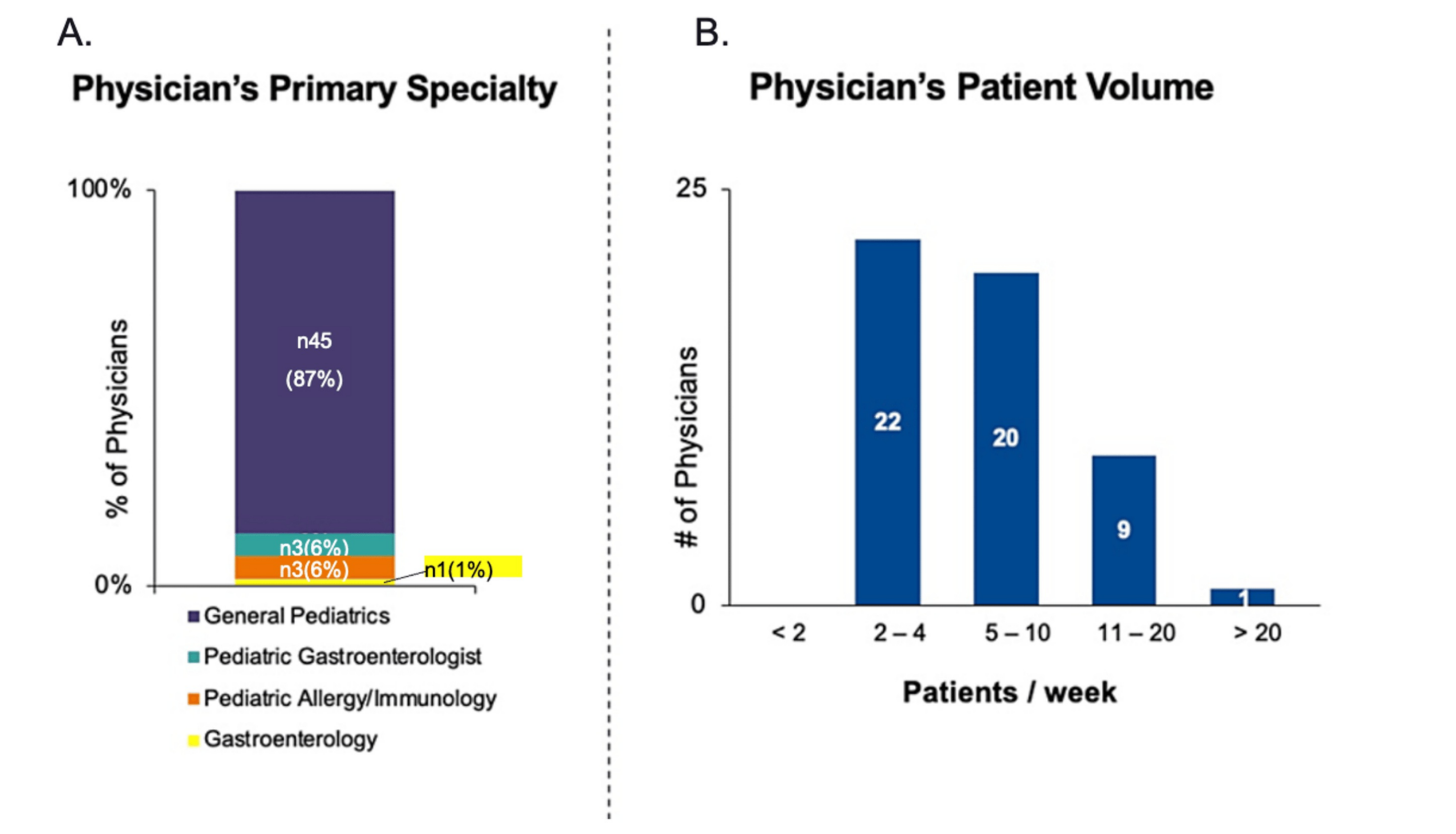
Physician Demographics: 2A- Physicians stratified by specialty. 2B- Number of cow's milk protein allergy (CMPA) patients seen per week by physicians

Of the 61 physicians eligible and collecting data, 52 (85%) completed both baseline and follow-up surveys using the interactive mobile survey application and were included in the final dataset. Nine physicians were excluded primarily for not submitting follow-up data. Most were general pediatricians (87%), with smaller numbers in pediatric gastroenterology and allergy/immunology (6% each), and one in gastroenterology (1%) (Figure 2A). Patient management frequency varied: 22 physicians saw two to four CMPA patients weekly, 20 saw five to 10, nine managed 11-20, and one handled over 20 (Figure 2B). No significant differences were found among groups (p>0.05).

Patient demographics and data collected

**Figure 3:**
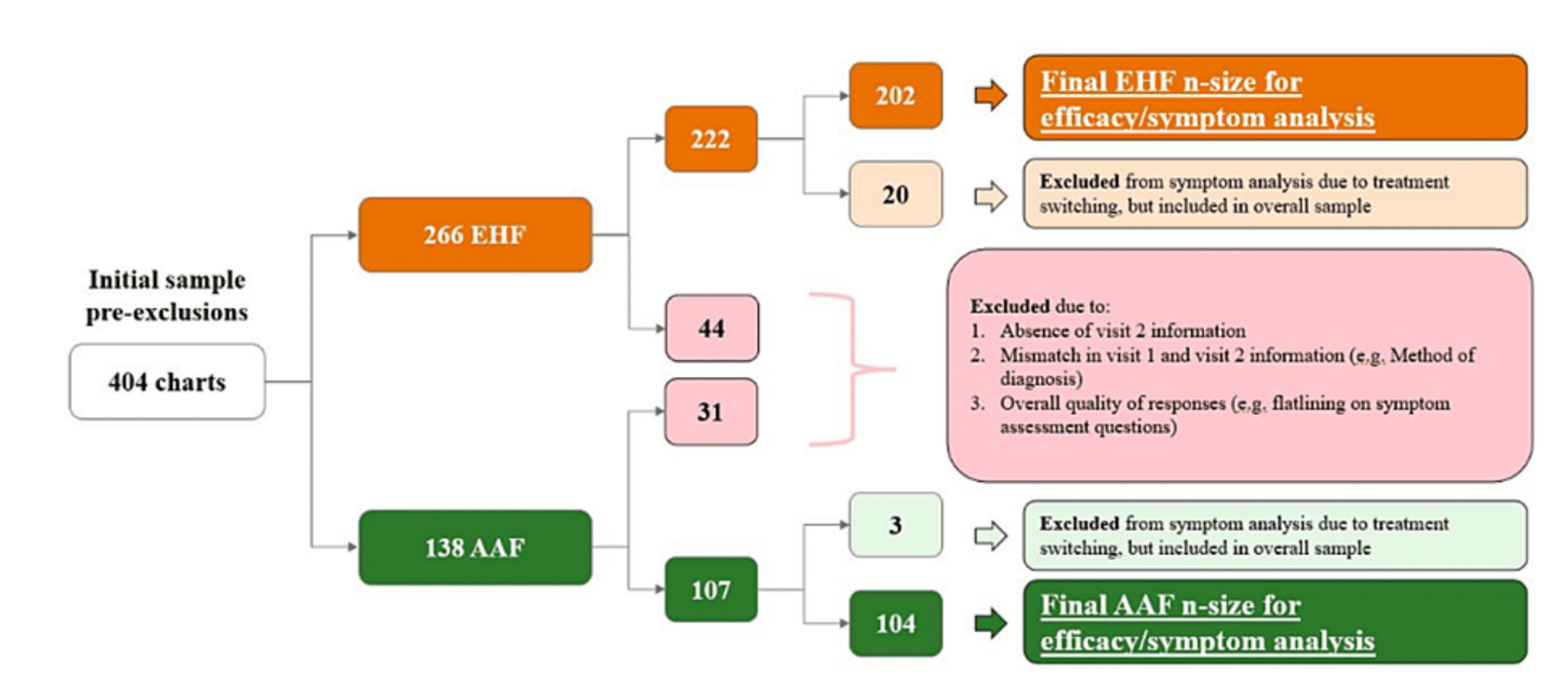
Patient inclusion flow sheet. EHF: extensively hydrolyzed formula; AAF: amino acid formula

**Figure 4:**
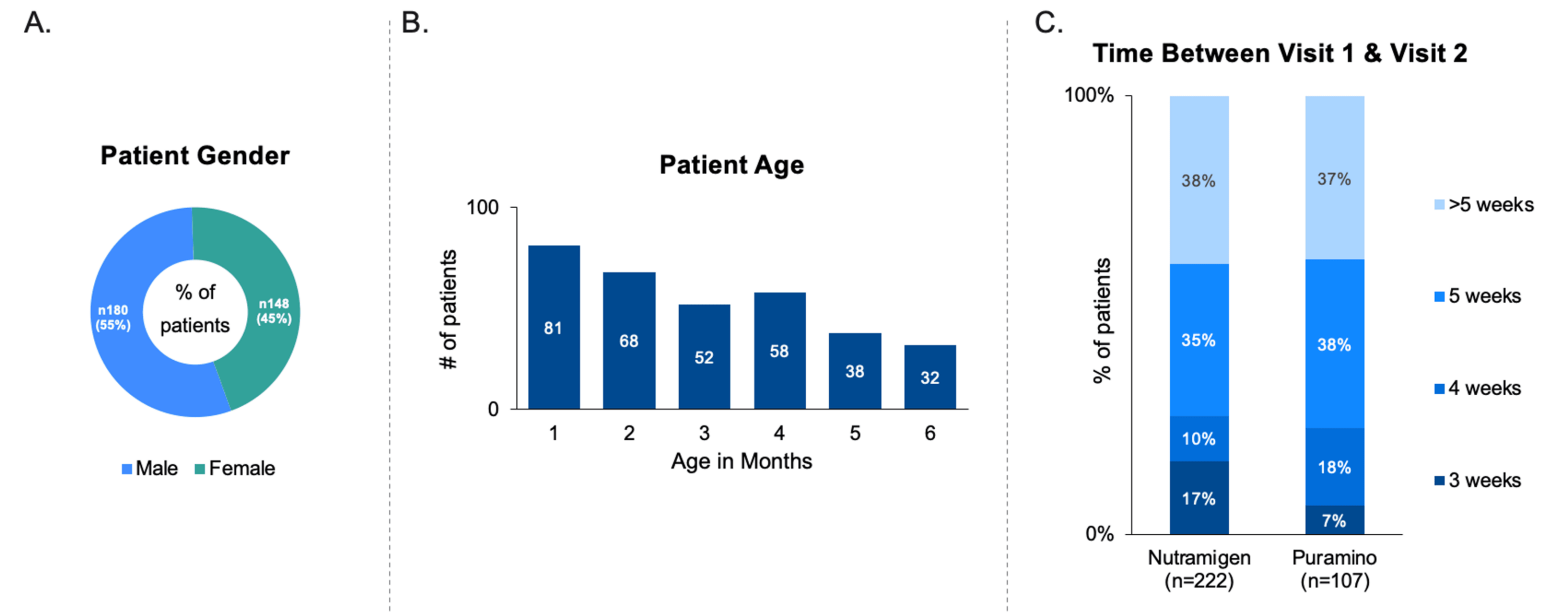
Patient Demographics: 4A- Gender demographics of patients. 4B- Age demographics of patients. 4C- Time between Visit 1 and Visit 2 stratified by number of weeks.

Data from 404 patients across 808 visits were collected, with 339 patients included in the final primary analysis: 222 using EHF and 107 using AAF. Figure 3 shows the stratification of included patient charts. Charts were mainly excluded for incomplete data or non-submission. Patient chart inclusion, categorized by physician specialty and formula type, is detailed in Table 3. No significant differences were found among groups (p>0.05). Patient demographics are illustrated in Figures 4A, 4B, and the duration between baseline and follow-up visits for both EHF and AAF cohorts is depicted in Figure 4C.

**Table 3:** Number of patient charts created on the interactive mobile survey application (used on baseline and follow-up visits) per physician specialty for patients on EHF or AAF. EHF: extensively hydrolyzed formula; AAF: amino acid formula

	Number of charts collected using the interactive mobile survey application for patients using EHF (n)	Number of charts collected using the interactive mobile survey application for patients using AAF (n)
General Pediatrics	194	88
Pediatric Gastroenterology	12	8
Pediatric Allergy/Immunology	11	6
Gastroenterology	5	5
Total	222	107

## Discussion

Our secondary analysis highlights the effectiveness of the interactive mobile survey application in real-time data collection within clinical settings. We found that 85% of physicians completed both initial and follow-up surveys using the application, demonstrating its potential to improve engagement and address common challenges in clinical research, such as time constraints and complex data management.

The high completion rate observed in our study addresses the significant barriers identified in previous literature, such as the extensive time commitments and challenges in managing patient data that often deter physician participation. Previous studies utilizing mobile applications in clinical research have allowed for increased insight into clinical reasoning and patient preferences [19,20]. Our findings suggest that the interactive mobile health application's usability and integrated privacy features significantly reduce these barriers, promoting greater physician involvement in clinical trials. This advancement is crucial given the historical reluctance of physicians to engage in research due to time constraints, administrative burdens, and privacy concerns [21-23]. 

Moreover, our study indicates that the interactive mobile health application's ease of use and efficient data management lowers barriers to physician participation. The findings underscore the benefits of integrating mobile technology in clinical settings to enhance research efficiency and engagement. Specifically, features like real-time data capture and a user-friendly interface in the interactive mobile survey application play a crucial role in facilitating these improvements.

Our study offers insights into the use of a mobile application by physicians in clinical research, but it's important to note its limitations. The small number of physician participants may affect the external validity of our results. There's also a potential selection bias, as technologically adept physicians might be more likely to participate. Additionally, the study did not extensively explore user satisfaction or feedback on the application’s functionality, which limits our understanding of its pros and cons. Furthermore, as this was a secondary analysis of existing data, no formal data monitoring process was implemented, which could affect data integrity and reliability assessment. Finally, the brief study duration of three to six weeks constrains our ability to evaluate the application's impact on long-term physician retention.

Our study suggests that mobile technology, exemplified by the interactive mobile survey application, has broader applications in clinical research beyond CMPA management, significantly enhancing efficiency and data quality. The high completion rates observed in our study highlight the potential for such applications to streamline data collection, thereby boosting participation and improving future research outcomes. These results support the expanding integration of technology in healthcare and underscore the need for further exploration to maximize the benefits of mobile applications in clinical research settings.

## Conclusions

In conclusion, our study highlights the potential of mobile software to improve clinical research by addressing longstanding participation challenges. Future studies should capitalize on our findings to explore innovative solutions and develop more effective clinical research tools.
